# Virtual Reality Visual Training in an Adult Patient with Anisometropic Amblyopia: Visual and Functional Magnetic Resonance Outcomes

**DOI:** 10.3390/vision5020022

**Published:** 2021-05-11

**Authors:** Juraj Halicka, Michal Bittsansky, Stefan Sivak, David P. Piñero, Peter Ziak

**Affiliations:** 1 Eye Clinic, Jessenius Faculty of Medicine, Commenius University in Bratislava, Kollárova 2, 036 59 Martin, Slovakia; euraay@gmail.com; 2 UVEA Mediklinik, Zelená 3739/1, 036 01 Martin-Priekopa, Slovakia; 3 Department of Biochemistry, Jessenius Faculty of Medicine, Commenius University in Bratislava, Mlynská dolina Ilkovičova 6, 842 15 Bratislava, Slovakia; michal.bittsansky@uniba.sk; 4 Neurology Clinic, Jessenius Faculty of Medicine, Commenius University in Bratislava, Kollárova 2, 036 59 Martin, Slovakia; stefan.sivak@uniba.sk; 5 Department of Optics, Pharmacology and Anatomy, University of Alicante, Crta San Vicente del Raspeig s/n, 03690 Alicante, Spain

**Keywords:** amblyopia, perceptual learning, occlusion therapy, patching, vision therapy

## Abstract

A case of an adult with anisometropic amblyopia who underwent a successful vision therapy program playing videogames in a virtual reality environment is described, reporting changes in conventional visual clinical data, as well as in brain activity. The patient was a 22 year old man on baseline examination that never previously wore correction for his anisometropia. After prescribing contact lens correction for the anisometropia and after 44 h of virtual reality-based vision therapy over a period of 1.5 years, the best corrected distance visual acuity (BCDVA) in the amblyopic eye improved from 0.05 to 0.5 (Sloan chart). One year after finishing the visual training, the BCDVA experienced a slight decrease to 0.4 (Sloan chart). Through the visual training, the patient gradually developed stereopsis. Likewise, changes were also detected after visual therapy on functional magnetic resonance imaging while the patient was viewing 2D and 3D stimuli. The preliminary results of this case show the potential of using virtual reality-based visual training as a treatment for adult amblyopia.

## 1. Introduction

Amblyopia is a visual condition with a prevalence of about 4% in children and 2% in adults. Adult people with only one functional eye can develop their working activities with normality, but many jobs require an active binocular vision with two fully functional eyes. Furthermore, for the amblyopic patient, the risk of becoming blind due to ocular trauma has been shown to be markedly higher than for the general population [1]. For this reason, the option of treating amblyopia in adults seems to be recommendable, promoting an improvement of the best corrected visual acuity and stereoscopic vision. However, the scientific evidence supporting the treatment of amblyopia in adults is still limited, especially studies showing the changes induced in the activity of the cortical area. In the current case report, the specific course of treatment using dichoptic training in virtual reality in a 22 year old amblyopic patient with hyperopia and anisometropia is described, evaluating changes in clinical data and in brain activity. It should be considered that most investigations on visual training in amblyopia were conducted in children who had high levels of neuroplasticity [2,3].

## 2. Case Report

### 2.1. Description of the Case

A case of a 22 year old male with hyperopia and late-diagnosed anisometropic amblyopia of the right eye is presented. The patient was diagnosed of amblyopia when he was 8, not following the recommendations from the ophthalmologist. Specifically, the patient did not wear the eyeglass correction required and was not compliant with occlusion therapy. No personal history of eye trauma or previous surgery was reported. At the beginning of the visual training, the patient was 22 years old, and he was studying medicine, being highly motivated in performing visual training as he wanted to become a surgeon. Visual training was irregular, adapted to a medicine student’s life pattern, consisting of 44 h of virtual reality-based training over a period of 1.5 years.

At the initial examination, his best corrected distance visual acuity (BCDVA) was 0.05 and 1.2 (decimal scale, Sloan chart) in the right and left eyes, respectively. Manifest refraction examination provided the following results: RE: +5 D Sph +2.5 D Cyl x 47° and LE: Plano. Worth’s four dot test showed a clear dominance of the left eye, without fusion of images. Stereopsis measured with the Wirt test was absent. Anisometropic hyperopia on the RE was corrected with a contact lens, +6 D Sph +2.25 D Cyl x 50°, with no visual improvement seen at 2 months after wearing the contact lens. Correction with glasses was not possible due to the presence of a not well tolerated aniseikonia. Accordingly, the visual therapy was continued.

As the vision therapy was prolonged over a long time, two different virtual reality head mounted displays were used, and different video games were used according to the evolution of the technology. In the beginning, dichoptic visual training was performed using the beta version of the computer game Diplopia Game (Vivid Vision, San Francisco, CA, USA), which was run in the Oculus Rift OC DK2 virtual reality head-mounted display (Oculus VR, LLC, Irvine, CA, USA). The OC DK2 was equipped with an AMOLED display (5.7′′ diagonal, resolution of 960 × 1080 pixels per eye), with a 100° field of view, mounted with an accelerometer, gyroscope, and magnetometer sensor for a positional tracking system. After 18 h of training, a newer model of Oculus Rift HD (1080 × 1200 pixel per eye resolution, 110° field of view) was used. The virtual reality head-mounted display Oculus Rift was connected to a PC system (Intel i5, 3,4 GHz, 8 GB RAM, Nvidia GeForce 970GT 4 GB).

**Figure 1:**
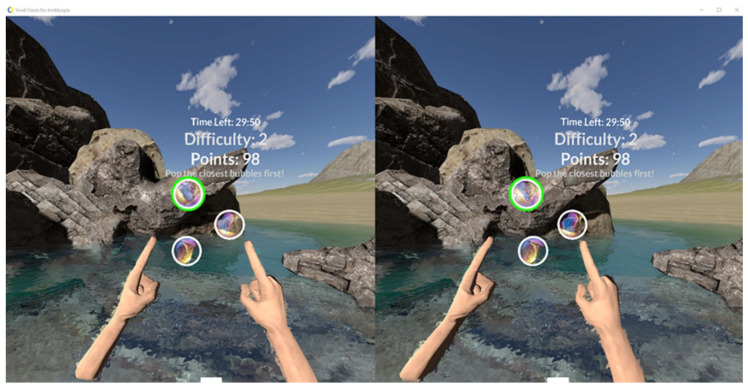
Bubbles game. Patient has to pick circles from the highest disparity continually to the lowest disparity. Different game difficulty levels are used. Both arms can be used by the patient to choose different bubbles.

Visual therapy was performed in dichoptic binocular settings; parts of the image shown in both eyes were different. For example, in the space game, the spaceship was visible only with the fixating, eye whereas colorful gates and asteroids were only visible with the amblyopic eye. As some objects were seen with the amblyopic eye and others were seen with the fellow eye, the game forced the brain to use both eyes together to play. More static games with different disparity setting were used, for example, bubbles, where the patient had to pick the highest-to-lowest disparity level of bubbles (Figure 1).

### 2.2. Vision Therapy

During the first month, the therapeutic procedure consisted of visual training using the Oculus Rift virtual reality helmet two times a week using the Diplopia Game beta version (VividVision, San Francisco, CA, USA), with a total duration of 8 h. Results were very encouraging and, after the first month of training, an improvement in the BCDVA of the amblyopic eye to 0.2 was observed (Sloan chart). The patient also perceived a subjective feeling of improvement, with a deep-stereo perception that he had not previously experienced. The patient then trained for another 10 h, without any further additional change. The follow-up of this patient was lost for 2 years after finishing this part of the vision therapy. Furthermore, the patient acknowledged afterward wearing the contact lens occasionally during this period.

Two years after the initial examination, the patient came to our consultation to check if the visual gain he had obtained was maintained. His BCDVA (+6 D Sph −2.25 D Cyl x 55°) on this examination was 0.32 in the right eye and 1.20 in the left eye. The patient reported perceiving four objects in the Worth test, although the top red rhombus was only seen faintly. The stereopsis measured with the Wirt test was 63”. The patient was recommended to wear full time correction again with contact lens in the amblyopic eye, being rechecked 3 months afterward, obtaining the same values of BCDVA and stereopsis. At this moment, a functional magnetic resonance imaging (fMRI) scan using 2D and 3D stimuli was also performed.

**Figure 2:**
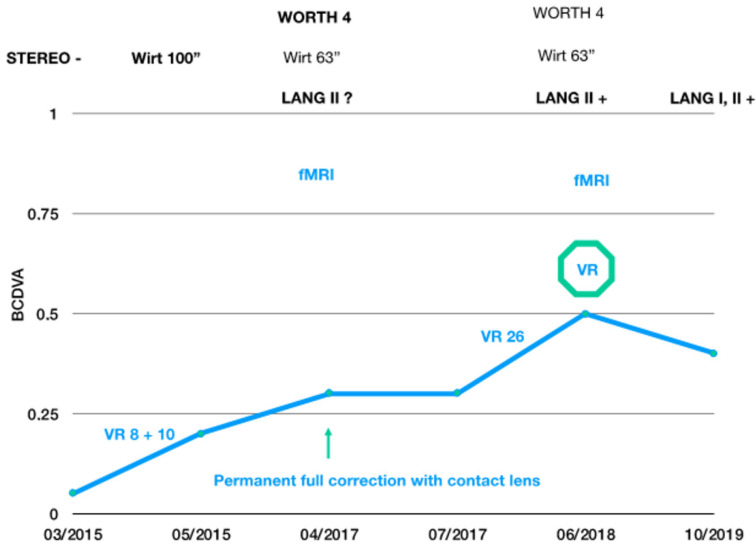
Summary of the visual training scheme. Time points are recorded on the *x*-axis, gains in BCDVA are recorded on the *y*-axis, and changes in stereopsis are recorded on top of the scheme. VR signs with different numbers denote how much time the patient spent in visual training in VR in the clinic. fMRI denotes when functional magnetic resonance imaging was performed, i.e., before and after VR therapy.

After this, the patient completed 26 sessions of 1 h of dichoptic training (a total of 44 h of training from the beginning). Worth’s four dot examination confirmed the presence of fusion. He could not see the pictures on the Lang I card, but he recognized the car and the moon in the Lang II test. Sixteen months later, his BCDVA was stable around a value of 0.4 (Sloan), with positive Lang I and II tests (Figure 2).

### 2.3. fMRI Testing

The patient was asked to wear polarized glasses to be examined with the Titmus 3D test when placed in the MRI device. A paradigm of 20 s was used. Each area (white background with a cross, Titmus 2D test, Titmus 3D test) was shown to the patient for 20 s, during which the BOLD-type fMRI brain activity was scanned. This procedure was repeated a total of six times, with randomized swapping of individual areas while scanning brain activity. In total, 18 baseline/Titmus 2D/Titmus test 3D changes were evaluated. The results were processed by the FSL system and analyzed by FEAT. Artefact suppression was provided by the ICA/AROMA system (Developers: fMRIB Analysis Group).

**Figure 3:**
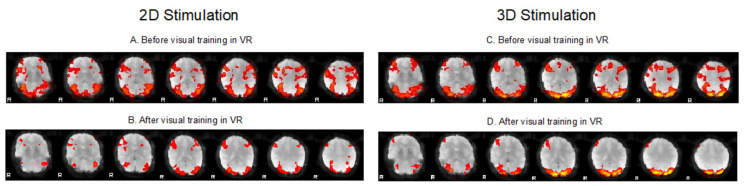
fMRI activation voxels showing activity in specific parts of the brain. (**A**,**B**) Comparison of fMRI before and after virtual reality-based training using a 2D stimulus (brain axial section set). The paradigm involved changing the Titmus 2D test and fixation image. See decreased activity in the frontal, parietal, and occipital lobes after treatment. (**C**,**D**) Comparison of fMRI before and after virtual reality-based training using a 3D stimulus (brain axial section set). The paradigm involved changing the Titmus 3D test and the fixation image. See decreased activity in frontal, parietal, and occipital lobes after treatment.

The result obtained after full time correction and 26 h of visual training activity is shown in the graph of fMRI visual cortex activity change (Figure 3). Figure 3B,D show reduced activation in the patient’s visual cortex compared to pretreatment examinations. Moreover, there is a visible difference in activity between 2D and 3D simulation. The dominant activation of the visual cortex in the occipital lobe persists.

## 3. Discussion

The patient evaluated in the current report was diagnosed of amblyopia at 8 years of age, when occlusion therapy was no longer effective due to limited tolerance to and compliance with this type of treatment. He did not wear the required eyeglass correction either. In summary, after 44 h of training, the left eye BCDVA improved to 0.5 (Sloan). Subjectively, he began perceiving 3D vision; in his own words, “... when I put on contact lenses, I felt the world become plastic.” Objectively, he began to see all 3D objects on Lang cards 1 year after finishing the visual therapy. This suggested that some changes were occurring in his visual cortex. Some months after finishing the training, the VA experienced a loss of around one line, but the patient gained stereovision, as tested at close distance with Lang stereo cards. A better outcome may have possibly been obtained by combining monocular visual training in a perceptual learning environment until achieving a value of 0.5–0.6 of visual acuity and treatment of interocular suppression with dichoptic training as recommended according to the most recent investigations [2].

This patient was one of our first adult patients undergoing visual training in a virtual reality environment. The value of this case is in showing the improvement of an adult amblyope trained using virtual reality-based vision therapy, with a follow-up of 5 years to check the stability of the outcome obtained. In adults, scientific evidence of the effectiveness of the treatment of amblyopia is scarce [3,4]. Recovery of normal visual functions is thought to be almost impossible after the critical period ends, i.e., after 8 years of age in children. However, there exist several animal and human studies that show visual pathway plasticity even after the critical period has passed, being patients who lost vision in the “good” eye with an improvement in the amblyopic eye being clear examples of this [5].

In anisometropic amblyopia, morphologic changes on MRI have been described [6], as well as changes in resting-state fMRI [7]. Functional magnetic resonance changes in the visual cortex during virtual reality-based vision therapy have not been previously described. During the stimulation of both 3D and 2D images, an overall reduction in activity in the visual cortex after vision therapy was observed. Several interpretations of these outcomes can be performed. This may be interpreted as a requirement of a lower brain activity to recognize both 2D and 3D images, leading to image perception and interpretation with less effort. Li and colleagues [8] performed an analysis of the retinotopic mapping and spatial frequency adaptation effects in the amblyopic cortex, finding a significant correlation between fMRI response and the magnitude of the adaptation effect. The authors suggested that a reduced adaptation may be a consequence of the reduced response to different stimuli reported for amblyopic eyes. Likewise, Thompson et al. [9] found that dichoptic viewing slightly reduced the BOLD response magnitude in amblyopic eye retinotopic regions in V1 and reduced the time to peak response. However, in the case described, peak activity did not seem to decrease, with the activity located in specific brain sites. Therefore, improved stability of fixation may explain many of these features of the fMRI after training. Future studies with large samples should be conducted to understand better the neural impact of this type of visual training in adults, confirming and providing a more consistent explanation for these preliminary findings.

In summary, neuroplasticity is still present in adult amblyopic patients, and visual recovery might be achieved with active visual training using the appropriate environments and stimuli [10]. The use of virtual reality has shown potential for increasing visual acuity in adults with anisometropic amblyopia, with an associated improvement in stereopsis [11]. Likewise, the use of other types of vision training programs has also shown a potential benefit in amblyopic adults [4,12,13]. Therefore, our results are consistent with those reported in previous experiences of vision training in adult amblyopia. Future studies should confirm in significant samples of adult amblyopic patients the potential benefit of using virtual reality environments over other modalities of vision training. Likewise, the impact of these findings in the refractive surgery field should be assessed further, considering the potential synergistic effect of the surgical procedure that leads to a change in the retinal image and an optimization of ocular optics [14,15], as well as the cortical activation induced by the virtual reality-based visual training.

## 4. Conclusions

These preliminary results show the potential of using virtual reality-based training as a treatment for adult amblyopia, while always being matched with the best possible correction of refractive error. Binocular dichoptic visual therapy in virtual reality was the only patient’s treatment option applied in this case after appropriate refractive correction. The exercise provided not only visual acuity improvement in the amblyopic eye but also improved stereopsis. This method of treatment of adult amblyopia requires patient cooperation and patience, with the possibility of improving the quality of life of adults who have not foreseen this possibility. A controlled clinical trial needs to be conducted to confirm these preliminary findings, as well as compare them with other types of exercises. In addition, our results suggest that some degree of spared cortical plasticity in the visual cortex can be “released” in the adult brain; however, more research is needed to better understand the real impact of this modality of treatment in adults.
